# The Spectrum of clinical manifestations in newborns with the COQ4 mutation: case series and literature review

**DOI:** 10.3389/fped.2024.1410133

**Published:** 2024-09-27

**Authors:** Pianpian Pan, Na Zhou, Yi Sun, Zhengrong Chen, Jin Han, Wei Zhou

**Affiliations:** ^1^ Nenoatal Intensive Care Unit, Guangzhou Wowen and Children’s Medical Center, Guangzhou Medical University, Guangzhou, China; ^2^ Heart Center, Guangzhou Wowen and Children’s Medical Center, Guangzhou Medical University, Guangzhou, China; ^3^ Pathology Department, Guangzhou Wowen and Children’s Medical Center, Guangzhou Medical University, Guangzhou, China; ^4^ Prenatal Diagnostic Center, Guangzhou Wowen and Children’s Medical Center, Guangzhou Medical University, Guangzhou, China

**Keywords:** COQ4, primary coenzyme Q10 deficiency, newborn, CoQ10, mitochondrial disorder

## Abstract

**Background:**

Coenzyme Q10 (CoQ10) plays an important role in the electron transport chain within the human mitochondrial respiratory chain. The manifestations of this deficiency exhibit a diverse range. This study investigates the clinical manifestations of primary coenzyme Q10 deficiency in neonates with the COQ4 mutation to improve the diagnosis of the disease and the prognosis through targeted treatment.

**Methods:**

We report 4 patients with primary coenzyme Q10 deficiency by COQ4 variants in neonates. A comprehensive literature search and review for original articles and case reports with COQ4 mutation published from January 1989 to November 2023 was performed through Pubmed. We review clinical manifestations, diagnostic approaches, and treatment monitoring in these and 20 previously reported patients.

**Results:**

Within the cohort of four cases examined, three females and one male were identified from two distinct families. Specifically, case 1 and 2 consisted of monoamniotic twins. Cases 3 and 4 were siblings. A comprehensive review of 20 cases involving neonatal-onset COQ4 mutation was conducted. Half of the cases are Chinese. There was no statistically significant difference in the mortality between Chinese (9/12, 75%) and other regions (11/12, 91.7%) (*P* = 0.27). The survival time for the 24 cases was 60.0 ± 98.0 days (95% confidence interval CI: 0–252.0 days). The incidence of prenatal abnormalities in preterm infants was significantly higher than that in full-term infants (66.7% vs. 16.7%, *P *= 0.02). Hyperlactatemia was one of the most common manifestations, accounting for 75% of cases (18/24). Twenty of the 24 cases were diagnosed by whole exome sequencing. Only 9 patients received exogenous coenzyme Q10 treatment, and all the 4 surviving patients received coenzyme Q10 supplementation.

**Conclusion:**

The prognosis of COQ4 mutation in the neonatal period indicates a low survival rate and an poor prognosis. This may be due to the incomplete understanding of the mechanism of how COQ4 gene defects lead to coenzyme Q10 deficiency and why CoQ10 supplementation does not respond well to treatment. To improve the diagnostic rate, in addition to genetic testing, mitochondrial functional verification should be prioritized in southern China, where the incidence is relatively high. It will facilitate more in-depth mechanistic studies.

## Introduction

Primary coenzyme Q10 deficiency is an autosomal recessive disorder that has been diagnosed in only about 300 patients worldwide to date (1). Primary CoQ10 deficiency affects multiple systems. As a result, the clinical presentation lacks specificity and is difficult to diagnose. Coenzyme Q (CoQ) is a class of endogenously synthesized redox-active lipids found in all intima, plasma membrane, and serum lipoproteins (2). The molecule is composed of a benzoquinone ring as the head group and a polyisoprene chain that inserts into the phospholipid bilayer (3); its length varies between species (4, 5). In humans and yeast, CoQ10 is composed of ten isoprene units and plays a vital role in the mitochondrial electron transport chain (mETC). It facilitates the transfer of electrons from Complexes I and II to Complex III, ultimately resulting in the development of an electrochemical gradient that propels ATP synthase towards the production of ATP. Moreover, it partakes in pyrimidine biosynthesis and apoptosis regulation, contributing significantly to eukaryotic cells (6–9). Mutations in *PDSS1, PDSS2, COQ2, COQ4, COQ5, COQ6, COQ7, COQ8A, COQ8B, and COQ9* genes have been found to cause primary coenzyme Q10 deficiency in humans (10). *HPDL* mutations may impair mitochondrial CoQ10 synthesis (11).The primary CoQ10 deficiency caused by *COQ4* variants was termed primary CoQ10 deficiency-7 (COQ10D7).

Due to the increasing popularity of second-generation gene sequencing, more cases are being diagnosed and researched, however, the high cost of genetic testing, along with the extended testing cycle and involving considerations of personal privacy (In some cases, parents may be reluctant to consent to testing due to concerns that abnormal results could lead to marital problems), means that numerous cases remain undiagnosed (12). Primary coenzyme Q10 deficiency is considered to be one of treatable mitochondrial disorder, and patients have been shown to benefit from coenzyme Q10 supplementation (13, 14). Therefore, early diagnosis and treatment for primary coenzyme Q10 deficiency are critical.

Our objectives in this case series and contemporary literature review were to enhance the understanding of neonatologists, geneticists and obstetricians regarding this disease in the early days, to investigate methods to improve the rate of diagnosis, and to advocate for further in-depth research on the mechanism involved.

## Methods

Four cases of *COQ4* gene mutation diagnosis from February 2020 to June 2022 at Guangzhou Women and Children's Medical Center were retrospectively summarized. Data collected included time of onset, sex, area, genotype, labor history, clinical presentation, biochemical examination, ancillary examination, and brain function assessment. The parents of case 2 consented to a local autopsy of the heart tissue, which was fixed with 4% neutral formalin, underwent routine dehydration, paraffin embedding, sectioning, Hematoxylin-eosin(HE) staining, and immunohistochemical light microscopy. Cases 2, 3, and 4 underwent a trio WES (whole exome sequence) test with the informed consent of the parents. The trio WES was conducted with an average coverage of 200X. The clinical context of these cases included decreased crying, decreased movement, cyanosis, and even severe hyperlactatemia, cardiogenic shock, and sudden, unexplained death. Genomic DNA was extracted from the peripheral blood and subsequently fragmented, spliced, amplified, and purified. The DNA library was then prepared by hybridization capture, and the exon and peripheral intron regions of 20,099 genes in the human whole exome were subsequently detected by high-throughput sequencing (20 bp). The sequencing data were then compared with the human genome hg19 (GRCh37) reference sequence, and the coverage of the target region and sequencing quality were evaluated. The pathogenicity of the variant was classifified according to standards and guidelines of the American College of Medical Genetics and Genomics. The variants suspected to be of clinical significance were confirmed by Sanger sequencing. The ratios of mosaic mutations in the peripheral blood were calculated using BioEdit software, which analyzed the Sanger sequencing results.

**Figure 1:**
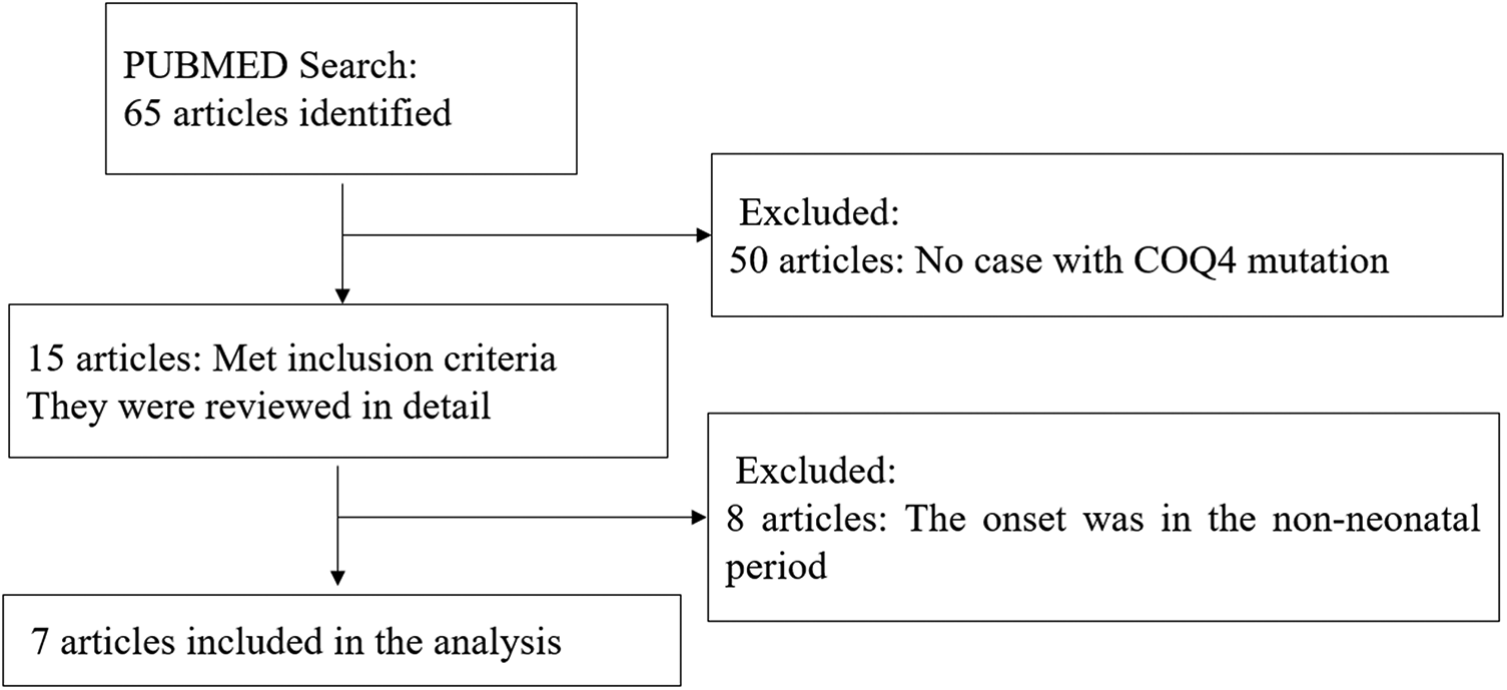
Flow chart showed identification and selection and cases having COQ4 mutation.

A comprehensive literature search and review for original articles and case reports with *COQ4* mutation published from January 1989 (15) to November 2023 was performed through PubMed. The main objective was to identify information on the spectrum of clinical manifestations and diagnosis of *COQ4* mutation. More specifically, data on sex, nationality, genotype, CoQ10 supplementation, and survival time were extracted when available. Two authors independently conducted the literature search using keywords *COQ4*, Primary coenzyme Q10 deficiency, and CoQ10. The abstracts of all selected articles were reviewed, and references from retrieved articles were used to identify other relevant sources. To focus on *COQ4* mutation with neonatal-onset, only cases with neonatal-onset primary Coenzyme Q10 deficiency were included (Figure 1).

### Statistical analyses

All data were analyzed using descriptive statistics, with values reported as the mean ± SD (standard deviation). Rates were compared using the chi-square test, with a *P-*value < 0.05 considered as statistically significant. Overall survival was calculated and presented as Kaplan–Meier curves. Statistical analyses were conducted using IBM SPSS statistics version 24.

### Result

#### Case series of *COQ4* mutation

**Figure 2:**
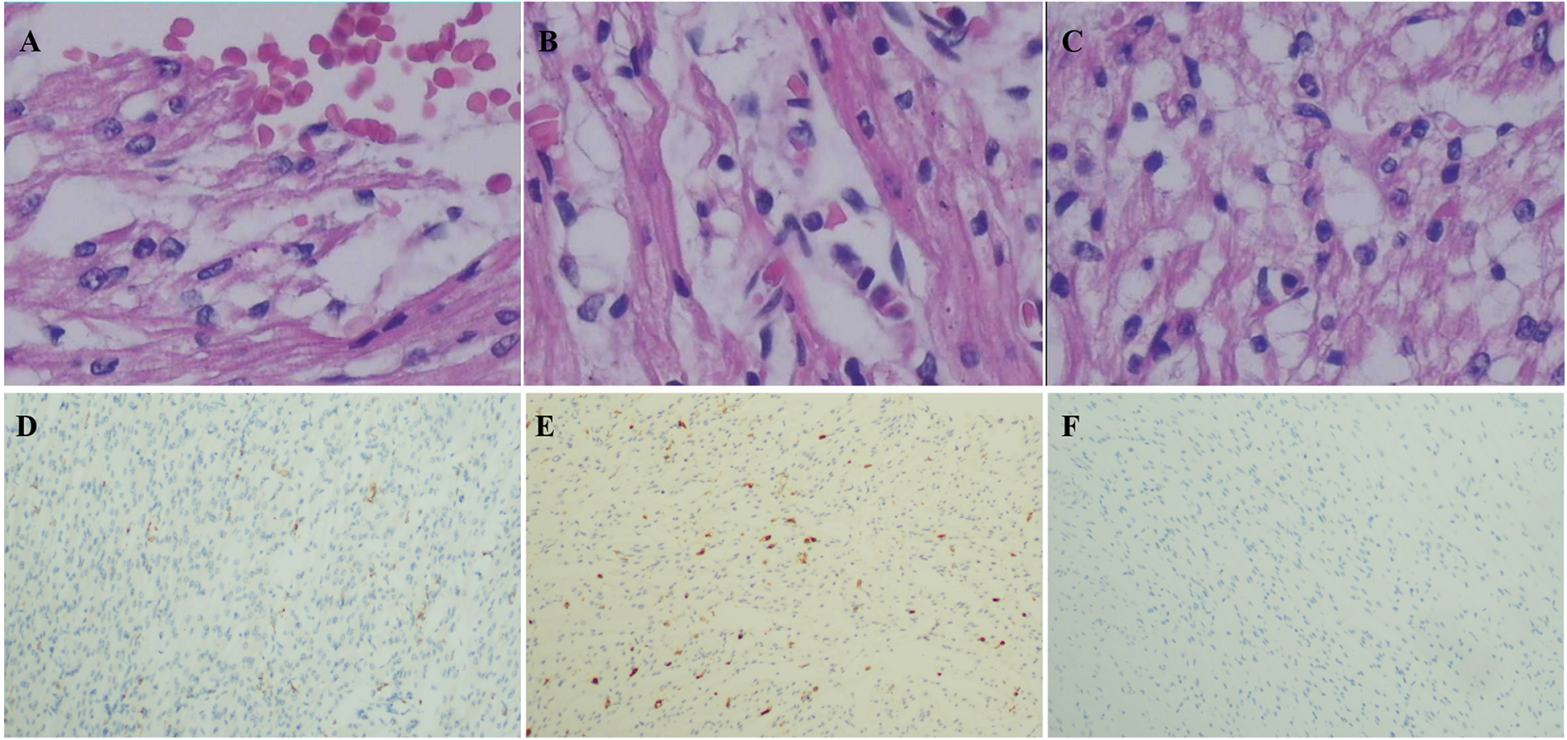
Pathological of myocardial tissue in case 2 after autopsy HE staining showed: **(A)** vacuole-like changes, interstitial porosity and edema, and punctate bleeding. **(B)** Myocardium vacuolated, interstitial loose edema, scattered lymphocyte infiltration; **(C)** cardiomyocytes were vacuolated and interstitial edema was observed. Immunohistochemistry: **(D)** CD68 (+), **(E)** LAC (+), **(F)** CMV (−). Microscope magnification 40*10 **(A**–**C)** and 40 **(D**–**F)**.

The four cases originated from two families with ancestral ties to Chaoshan, Guangdong Province (Table 1). They were all non-consanguineous marriages. Among them, cases 1 and 2 were monoamniotic twins born late preterm and small for gestational age, while cases 3 and 4 were full-term infants appropriate for gestational age. All four cases had underwent regular prenatal examinations with no abnormal fetal development detected, and there was no history of birth asphyxia rescue. Newborn screening tests for neonatal genetic metabolic diseases by tandem mass spectrometry were normal for cases 3 and 4. However, due to the rapid changes in their birth conditions, screening was not conducted for cases 1 and 2. Initial symptoms reported in the four patients included reduced crying, decreased movement, feeding difficulties, gradual muscle tone decline, apnea, cyanosis. As the condition deteriorated, hyperlactatemia and cardiogenic shock manifested. Plasmatic amino acids and acylcarnitines did not show significant findings, but urine organic acid analysis revealed elevated levels of lactic acid, pyruvic acid, or 2-OH glutaric acid. Cases 1, 2, and 3 developed cardiogenic shock, while none of the patients experienced liver and kidney dysfunction. Cardiac tissue of case 2 was examined postmorterm(Figure 2). The myocardial tissue observed during the autopsy of the child exhibited a grayish-red color and soft texture. Histological examination using HE staining revealed vacuolar degeneration of cardiomyocytes, individual necrosis, myocardial interstitial porosity, edema, and scattered lymphocyte infiltration (Figures 2A–C). Immunohistochemical analysis under light microscopy indicated intermyocardial edema degeneration, primarily interstitial lymphatic invasion, CD68 (+), LAC (+), CMV (−) (Figures 2D,E). WES was performed to confirm the diagnosis in the cases 2, 3, 4. The COQ4 gene sequencing for cases 1 and 3 is in the Supplementary Files. Case 1 died suddenly due to rapid changes in her condition, so no blood samples were collected for WES. Case 1 was diagnosed based on her monozygotic twin (Case 2) gene reporter when Case 2 presented with similar symptoms. Among the cases, Case 4 received a coenzyme Q10 supplement during the neonatal period and survived, while the other three patients succumbed during the neonatal period.

**Table 1:** Clinical data of case series in the study.

	Family 1		Family 2	
	Case 1	Case 2	Case 3	Case 4
Paternal geno	C.370G>A/P.G124s		C.370G>A/P.G124s	
Maternal geno	C.300-2A>G/P.?		C.370G>A/P.G124s	
Sex	Female	Female	Female	Male
GA(W)	36^+1^	36^+1^	39^+1^	39
BW (g)	1,700(<P3)	1,950(P3-P10)	2,850(AGA)	3,015(AGA)
Apgar score	8-9-10	8-8-9	10-10-10	10-10-10
Clinical manifestations	Dyspnea, cyanosis, hypotonia, feeding difficulties			
Heart faiture	Yes	Yes	Yes	Yes
seizure	No	Yes	Unkown	Yes
LAC(<2 mmol/L)	15	15	16.7	14.9
BS(mmol/L)	24.9	27.8	5.1	Norm
Hepatic, renal	NL	NL	NL	NL
Urine GC-MS	Not done	Not done	Not done	Lac, pyruvate↑
Blood amino acid analysis	Not done	Alanine, proline↑	NL	NL

GA, gestation age; BW, birth weight; AGA, appropriate for gestational age; LAC, lactate; BS, blood glucose; ↑, elevate; NL, normal.

**Figure 3:**
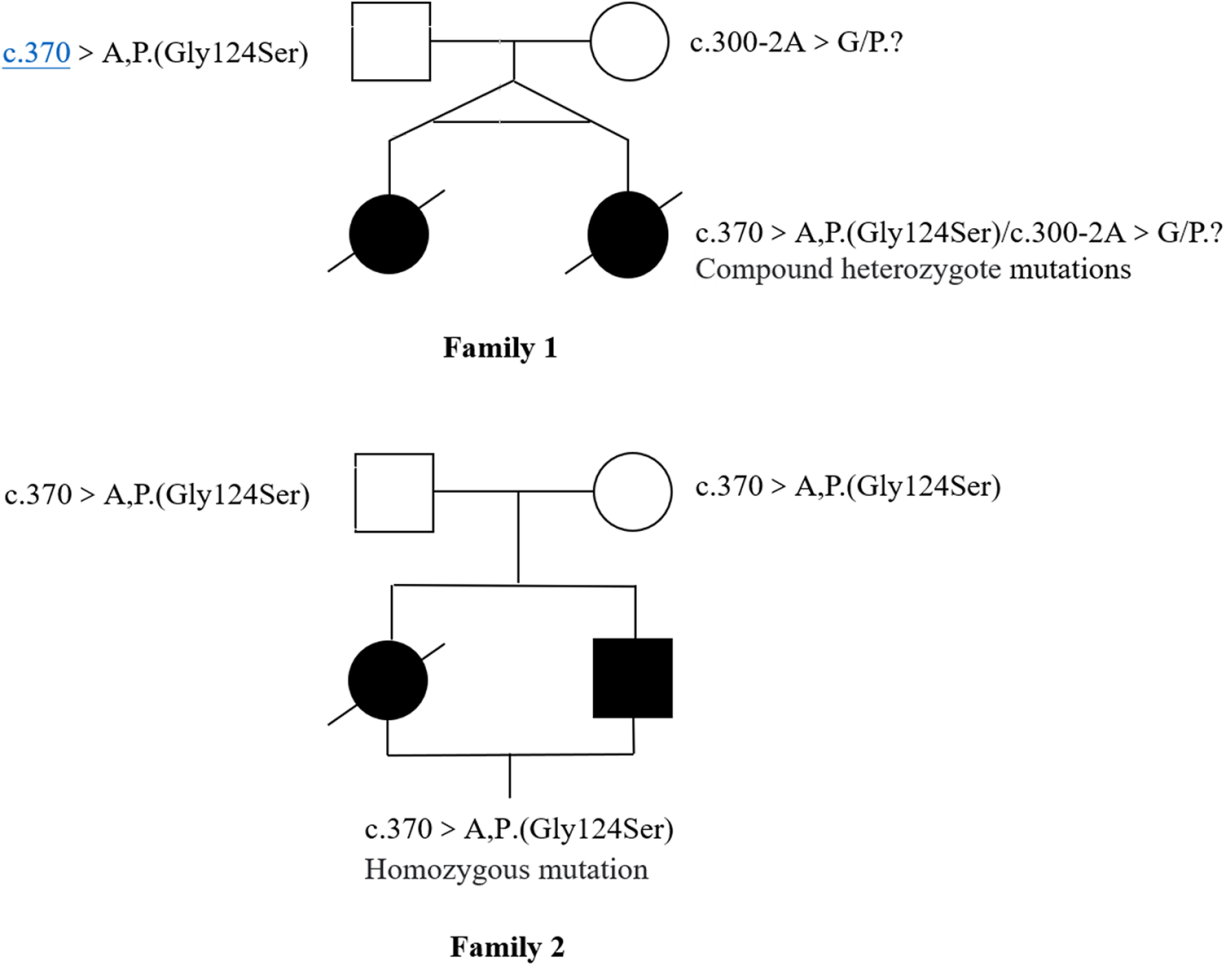
Pedigrees plot of four cases of primary coenzyme Q10 deficiency with COQ4 mutation.

Novel heterozygous variants were identified in case 1 and case 2 through whole-exome sequencing, specifically a pathogenic NM_016035.3:c.300-2A>G(p.?) variant in the COQ4 gene. This variant was classified as pathogenic based on the ACMG guidelines with an evidence class of PVS1 + PM2 + PM3. The mother carried the c.300-2A>G (p.?) mutation, which is located in the classical splice site region within intron 3. The father was found to have a heterozygous variation with c.370G>A (p. G124S) in the COQ4 gene. In case 3 and case 4, who are siblings, a homozygous c.370G>A (p.G124S) mutation was identified, which was confirmed to be inherited from the parental side through whole-exome sequencing. The diagnosis was established through whole-exome sequencing, as depicted in Figure 3.

**Figure 4:**
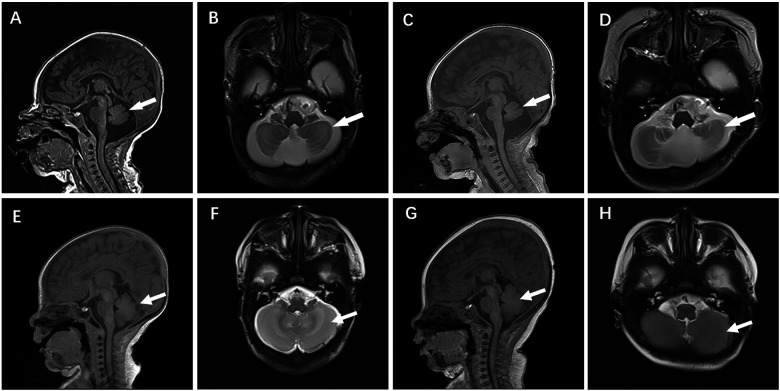
Cranial MR images of case 4 brain MR Images of the 2-week-old showed cerebellar hypoplasia on **(A)** (T1WI) and **(B)** (T2WI) images. At 2 months, **(C)** (T1WI) and **(D)** (T2WI) images of the same level showed aggravated cerebellar dysplasia. **(E**–**H)** displayed a normal skull image of the same age and sex, with the arrows indicating the cerebellum.

Case 4 was administered oral coenzyme Q10 treatment at four weeks of age, as detailed in Table 2. Throughout the treatment period, the patient underwent periodic brain MRI scans, EEG tests, and assessments of plasma coenzyme Q10 concentration. Initial brain imaging did not reveal any significant changes. However, after two months, Compared to boys his age, MRI scans indicated a more pronounced cerebellar dysplasia in the coronal T1-weighted and sagittal T2-weighted images, despite the coenzyme Q10 administration (Figure 4). The EEG showed epileptic wave ranging from localized to generalized. Phenobarbital has exhibited favorable results in treating neonatal seizures. During a stable phase, Case 4 contracted rotavirus, leading to a deterioration in the patient's condition, including respiratory failure, reduced myocardial contractility, and severe lactic acidosis (with normal lactic acid levels prior to the infection, but severe hyperlactacemia during the rotavirus episode). Interventions such as respiratory and circulatory support, along with an increased dosage of coenzyme Q10 (50 mg/kg), successfully reversed the fatal respiratory and circulatory failure, restoring lactic acid levels to normal. Plasma coenzyme Q10 concentrations were monitored throughout the treatment period, ranging from 1.52 to 4.91 umol/L (Reference value range is 0.46–1.85 umol/L). A decrease in concentration (1.65 umol/L) was noted during symptom exacerbation, followed by an increase (8.45 umol/L) after supplementation, leading to symptomatic improvement.

**Table 2:** Treatment and follow-up of case 4.

Time of threapy	Dose of CoQ10	CoQ10 in plasma (0.46–1.85 μmol/L)	Clinical Manifestation	Electroencephalogram (EEG)	Antiepileptic threapy
27 days after birth	10 mg/kg	1.52 μmol/L	Cyanosis seizures	Electrical seizures in occipital region	Luminal
55 days after birth	20 mg/kg	4.91 μmol/L	Physical inactivity, Weak crying Dysthelasia	Slight delay in maturity background activity	None
65 days after birth (rotavirus infection)	20 mg/kg	1.65 μmol/L	Increased seizures, lactic acid elevation	Lots of low-amplitude spike waves in occipital, parietal and temporal regions.	Levetiracetam Clonazepam
76 days after birth	40 mg/kg	8.45 μmol/L	Seizure improved, dysthelasia	Background activity normal. Numerous amplitude spikes/spikes	Clonazepam

#### Review of neonatal cases caused by COQ4 mutation

Out of 65 articles identified with keywords, 50 were excluded due to the lack of COQ4 mutation cases. Therefore, only 7 articles related to neonatal onset were eligible for in-depth review. There were 20 neonatal cases attributed to COQ4 allele variants in the case series and 4 from our case series, totaling 24 cases. The details of the sex, nation, genotype, and survival time are summarized (Table 3), the main clinical manifestations, diagnosis, and therapy are summarized (Table 4). The references in the Tables 3, 4 are from (16–22).

**Table 3:** Compiled data on sex, area, genotype and survival time in COQ4 mutation with neonatal-onset.

Refenrece	No.	Sex	Nation or area	GA	Prenatal test	Genotype, exon	Variation	Survival time
This study	1^F1^	F	Guangdong Of China	36 + 1 weeks	NL	c.370G>A/c.300-2A>G, 4/Intron3Het	Mis/Splice	0.42 days
	2^F1^	F		36 + 1 weeks	NL			2.13 days
	3^F2^	F		39 + 1 weeks	NL	c.370G>A/c.370G>A,4Hom	Mis	10 days
	4^F2^	M		39 weeks	NL			540 days(LFT)
Chung et al. (20)	5	M	Italy	Term	NL	c.433C>G/c.433C>G, 5/5Hom	Mis	0.17 days
	6	F	Japan	34 weeks	IUGR,HCM	c.421C>T/c.718C>T, 5/7Het	Non/Mis	1 day
	7^F3^	F	Austrian	32 weeks	CH	c.155T>C/c.521_523del, 2/5Het	Mis/Dele	3 days
	8^F3^	F	Austrian	34 weeks				2 days
Brea-Calvo et al. (19)	9^F4^	F	Caucasian–Hispanic	39 weeks	NL	c.245T>A/c.473G>A, 3/5Het	Mis	60 days
	10^F4^	F		40 weeks	NL			1.5 days
	11	F	Jewish	37 weeks	IUGR,HCM,CH	c.718C>T/c.718C>T, 7Hom	Mis	4 days
	12^F5^	F	Caucasian	Term	NL	c.197_198delinsAA/c.202G>C, 2/3Het	Mis	570 days
	13^F5^	F		38 weeks	NL			300 days
	14	F	Jewish	Term	NL	c.718C>T/c.718C>T, 7Hom	Mis	210 days
Sondheimer et al. (21)	15	M	Canada	Term	NL	c.23-33del11/c.331G>T; c.356C>T, 1/4Het	Dele/Mis	120 days
Yu et al. (16)	16	M	Hong Kong and Taiwan	38 weeks	IUGR	c.370G>A/c.402+1G>C, 4/Intron4Het c.370G>A/c.402+1G>C, 4/Intron4Het	Splice/Mis	240 days
	17	M		Term	NL		Splice/Mis	2.5days
	18	F		37 weeks	NL	c.370G>A/c.370G>A, 4Hom	Mis	270 days
	19	F		38 weeks	IUGR	c.370G>A/c.402+1G>C, 4/Intron4Het	Splice/Mis	1,460 days(LFT)
	20	F		Term	NL	c.370G>A/c.370G>A,4Hom	Mis	365 days
Ling et al. (18)	21	M	Hongkong	33 + 4w	IUGR	c.370G>A/c.533G>A,4/6Het	Mis	28 days
Lu et al. (17)	22^F6^	M	Fujian of China	39w	NL	c.370G>A/c.370G>A,4Hom	Mis	170 days
	23^F6^	F		Term	NL			1,460 days(LFT)
Hashemi et al. (22)	24	F	Iran	Term	NL	c.437T>G/c.437T>G, 5Hom	Mis	3,285 days(LFT)

F, female; M, male; ^F1^, represents family code; GA, gestation age; IUGR, intrauterine growth delay; HCM, hypertrophic cardiomyopathy; CH, cerebellar hypoplasia; NL, normale; Het, compound heterozygote; Hom, homozygote; Mis, missense; Non, nonsense; Dele, deletion; LFT, last follow-up time.

**Table 4:** Compiled data on the main clinical manifestations, diagnosis, therapy, death age in COQ4 mutation with neonatal-onset.

Refenrece	No.	Clinical manifestations											Diagnosis					Therapy				Death age
		Hypomyotonia	cardiomyopathy	Arrhythmia	Respiratory failure	Apnoeic	lactic acidosis	Renal fuction	Liver fuction	Seizure	Feeding difficulties	DD	WES	mECT activity	Clinical Definite	CoQ10 testing	Autopsy	Ventilator	I.V cardiac	Oral CoQ10	Antiepileptic	
This Study	1^F1^		√	√	√	√	√	NL	NL				√		√		√	√	√			10 h
	2^F2^		√	√	√	√	√	NL	NL	√			√					√	√			51 h
	3^F2^	√			√	√	√	NL	NL		√		√					√	√			10 days
	4^F2^	√	√	√	√	√	√	NL	NL	√	√	√	√			√		√		√	√	18 months (LFT)
Chung et al. **(**20)	5	√	√	√	√	√	√	NL	NL				√	√		√	√	√	√			4 h
	6	√	√		√	√	√	NL	NL				√	√			√	√				1 days
	7^F3^				√		√	NL	NL	√				√	√	√	√	√				3 days
	8^F3^				√		√	NL	NL	√			√	√		√	√	√				2 days
Brea-Calvo et al. (19)	9^F4^	√	√		√		√	NL	NL	√	√		√	√			√	√	√	√	√	2 months
	10^F4^		√		√	√	√	NL	NL	√					√		√	√	√		√	36 h
	11	√	√		√		√	NL	NL	√			√	√			√	√	√			4 days
	12^F5^	√	√		√			NL	NL	√	√	√	√					√				19 months
	13^F5^	√			√		√	NL	NL		√				√			√				10 weeks
	14	√	√	√	√	√		NL	NL	√	√		√					√		√		7 weeks
Sondheimer et al. (21)	15	√	√	√	√	√	√	NL	NL	√	√		√	√		√	√	√			√	4 months
Yu et al. (16)	16	√	√	√	√	√	√	NL	NL	√		√	√	√		√		√	√	√	√	8 months
	17		√		√	√	√	NL	NL		√		√					√	√	√		2.5 days
	18	√	√		√		√	NL	NL	√		√	√									9 months
	19	√	√		√	√	√	NL	NL	√		√	√					√		√	√	4 years (LFT)
	20	√						NL	NL	√	√	√	√	√		√				√		3 years 6 months
Ling et al. (18)	21	√	√		√	√	√	NL	NL				√				√	√	√			28 days
Lu et al. (17)	22^F6^	√	√	√	√			NL	NL	√	√		√					√				5 months 20 days
	23^F6^	√			√			NL	NL	√	√	√	√	√						√		4 years (LFT)
Hashemi et al. (22)	24	√			√			NL	NL	√	√	√	√					√		√	√	9 years (LFT)
Total	24	18	17	8	23	13	18	24	24	17	12	8	21	10	4	7	10	22	10	9	7	

Blank, not provided; DD, global development delay; WES, whole exome sequencing; mECT, mitochondrial electron transport chain; I.V, intraveous; NL, normal; LFT, last follow-up time.

#### Clinical manifestations of primary CoQ10 deficiency

**Figure 5:**
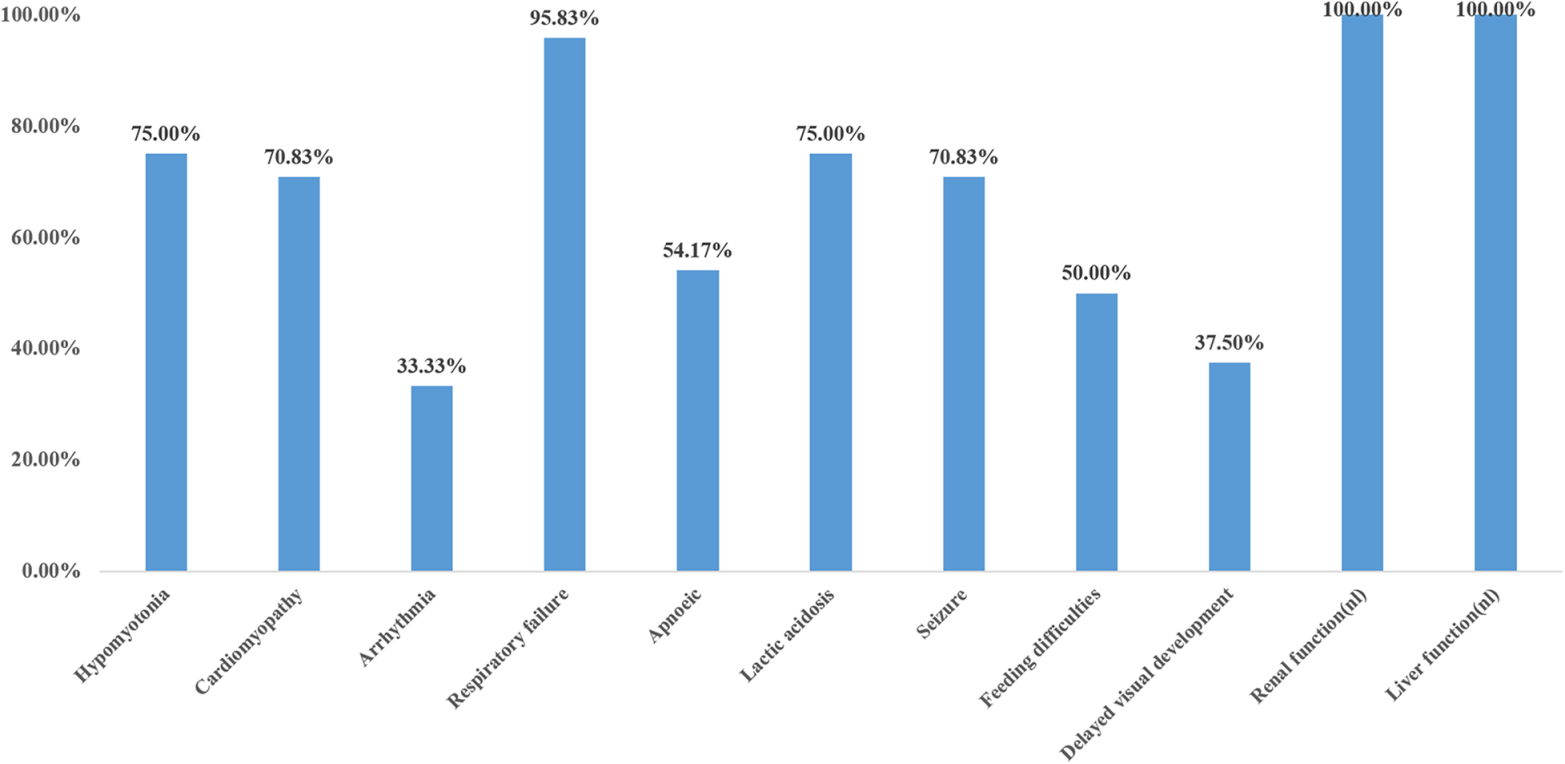
Clinical features of primary coenzyme Q10 deficiency in the neonatal (*N* = 24).

The main clinical manifestations of 24 cases were analyzed (Figure 5). Six cases were preterm infants, and 18 cases were full-term infants. Five cases (20.8%) had intrauterine growth restriction, two cases (8.3%) had cardiac hypertrophy, and two cases (8.3%) had cerebellar hypoplasia. Sixteen cases (66.6%) had normal prenatal examination. Four out of six preterm infants (4/6, 66.7%) and three out of 18 full-term infants (3/18, 16.7%) were found to have prenatal abnormalities. The incidence of prenatal abnormalities in preterm infants was significantly higher than that in full-term infants (66.7% vs. 16.7%, *X*^2^ = 5.4, *P* = 0.02 < 0.05). There was a statistically significant difference. Hypotonia was found in 18 out of 24 cases (75%), the remaining six cases were not mentioned in the case report, and all of them died in the early neonatal period (<4 days of age). Cardiomyopathy was present in 70.8% (17/24) of the patients, and 8/24(33.3%) had an arrhythmia. Among the other seven patients without cardiomyopathy, three survived beyond 3 years old, Almost all patients (23/24) had respiratory failure, which was not reported in the literature in case 20, who carried a homozygous c.370G>A mutation presented with generalized dystonia, feeding difficulties, and global developmental disability. In addition, hyperlactatemia was one of the most common manifestations, accounting for 75% (18/24), and all the neonates who died in the neonatal period had hyperlactatemia. All patients had normal liver and kidney functions. Standard newborn screening tests of cases 3, 4, 6, 7, 18, 19, 22, 24 were normal; the remaining cases were not done or provided. 70.8% (17/24) of the patients had epilepsy, and 50% (12/24) had feeding difficulties. 37.5% (8/24) of patients had global developmental disabilities, but their minimum survival time was 8 months.

#### Diagnosis and treatment of primary COQ deficiency

**Figure 6:**
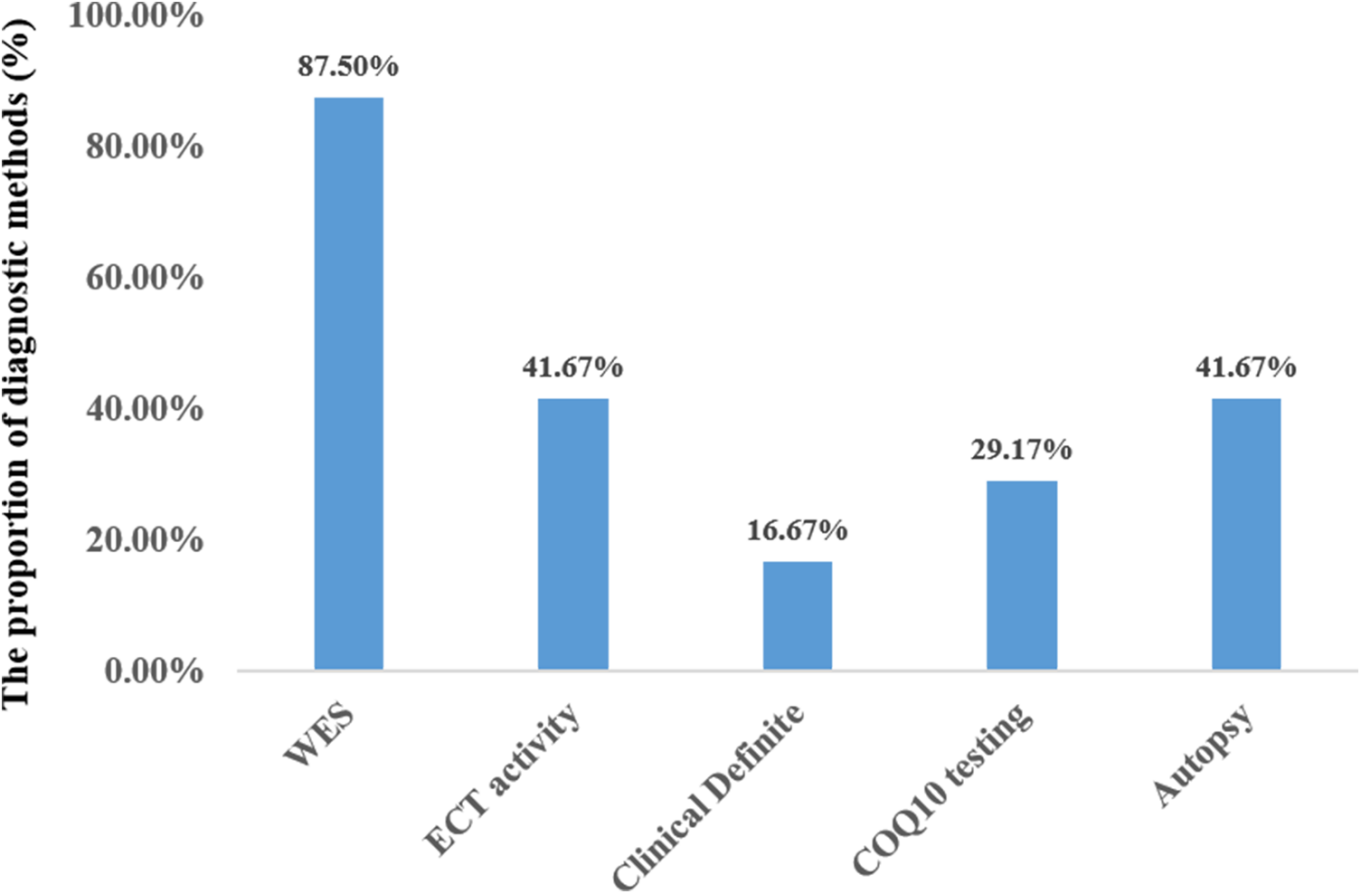
Diagnostic methods of primary coenzyme Q10 deficiency in the neonatal (*N* = 24).

Twenty-one out of the 24 cases were diagnosed through WES. Three patients were diagnosed based on genetic findings from their siblings, along with similar clinical symptoms. Less than half of the patients underwent testing for mitochondrial complex enzyme activity (10/24, 41.6%) and coenzyme Q10 concentration (7/24, 29.2%). In 10 out of the 24 cases, the patient's family agreed to autopsy (Figure 6). Treatment mainly included cardiopulmonary support, antiepileptic drugs, and exogenous coenzyme Q10. The majority of the patients required respiratory support (22/24), while a few needed intravenous inotropic drugs (10/24). Seven out of the 24 patients received antiepileptic therapy, and 4 of them experienced global developmental delay, except for 3 who passed away before reaching 4 months of age. Only 9 patients were administered exogenous coenzyme Q10 treatment, and all the 4 surviving patients received coenzyme Q10 supplementation.

#### Genotype and prognosis

**Figure 7:**
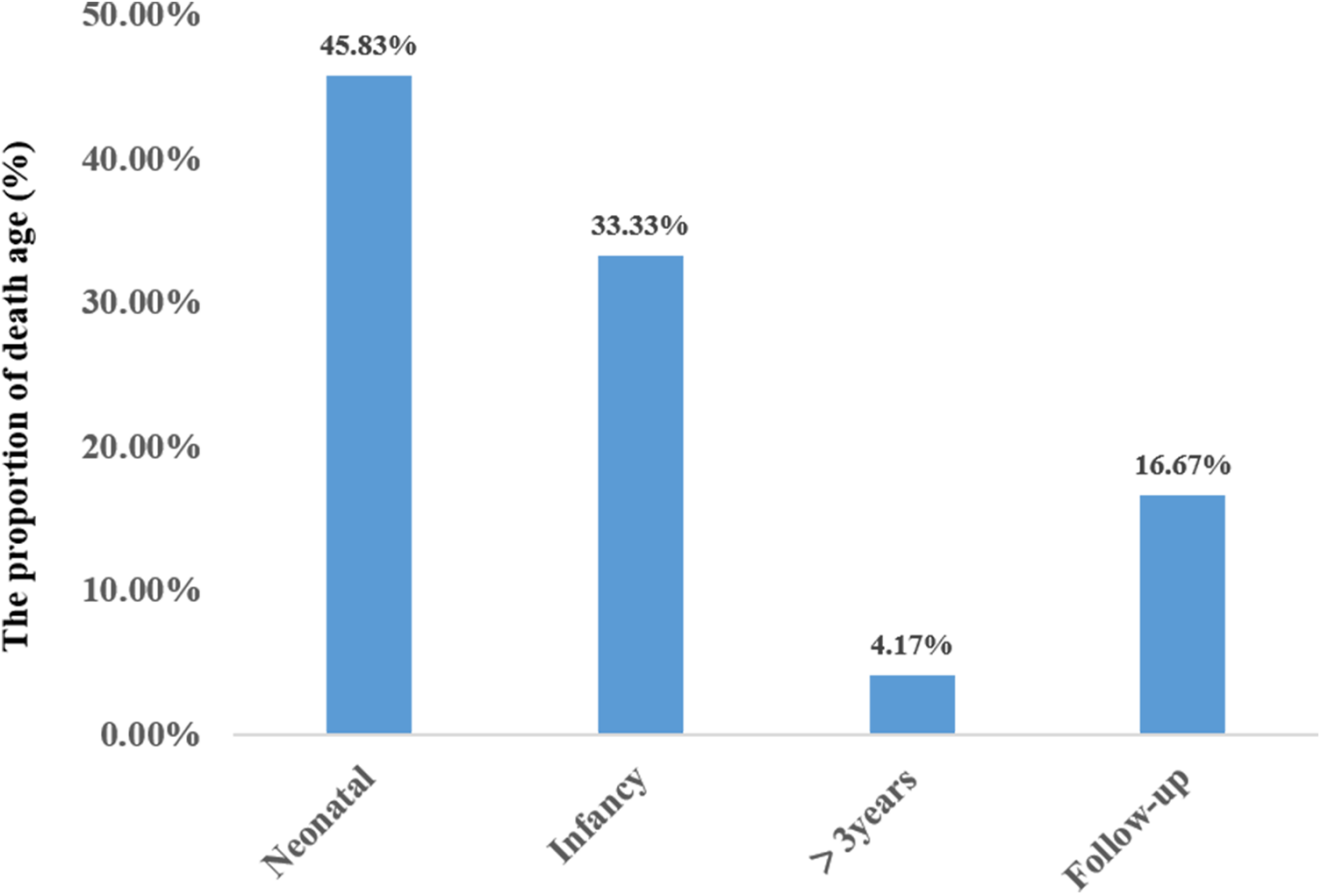
Age distribution at survival of primary coenzyme Q10 deficiency (*N* = 24).

A total of 24 neonatal-onset patients with COQ4 gene variants came from 18 families in 8 countries, of which 50% (12/24) were from China. There were 7 males and 17 females, with 10 compound heterozygous variants and 14 heterozygous variants. The rate of compound heterozygous variants in premature infants (6/6, 100%) was significantly higher than that in full-term infants (8/18, 44.4%) (100% vs. 44.4%, *X*^2^ = 5.7, *P* = 0.02 < 0.05), showing statistically significant difference. The mortality rate of complex heterozygous variants (13/14, 92.9%) was not significantly different from that of homozygous mutations (7/10, 70%) (92.9% vs. 70%, *X*^2^ = 2.2, *P* = 0.27 > 0.05). The mutation frequency was highest in exon 4 and lowest in exon 1 and 6. There was no statistically significant difference in mortality between Chinese patients (9/12, 75%) and those from other regions (11/12, 91.7%) (*X*^2^ = 1.2, *P* = 0.27 > 0.05). The types of mutations included missense mutations, nonsense mutations, deletion mutations, and splicing mutation, with missense mutation being the most common. The survival time for the twenty-four cases was 60.0 ± 98.0 days (95% confidence interval CI: 0–252.0 days). Eleven cases resulted in death during the neonatal period. Three cases (cases 19,23,24) survived beyond three years of age, with the mutation sites located in exons 4 and 5 (Figure 7).

## Discussion

The primary coenzyme Q10 deficiency caused by COQ4 gene variation in the neonatal period has characteristic clinical manifestations: hyperlactatemia, cyanosis, apnea, convulsions, feeding difficulties, cardiogenic shock, but no distinctive physical features. Similar to other genetic disorders, the presence of comparable cases in families is highly suspected. In these 24 cases, we observed varying degrees of cardiac, respiratory, and brain dysfunction among different patients. Although active treatment cannot cure secondary epilepsy and improve neurological damage such as DD (global development delay), CoQ10 treatment could prolong life expectancy. Throughout the disease progression, liver and kidney function remained normal, unlike the genetic variants associated with that renal involvement (*COQ8B, COQ2, COQ6, PDSS2*) (10). Brain dysfunction is notably pronounced in patients with COQ4 gene mutation. In patients treated with CoQ10, respiratory and cardiac functions remained stable, but the advancement of brain dysfunction was not reversed. These findings are intriguing as they differ from other common genetic defects: (1) Patients did not have any dysmorphic features; (2) Neonatal disease screening results are normal; (3) Most individuals were born full-term, had normal birth and prenatal examinations; (4) Rapid changes and disease progression occurred after birth. Primary coenzyme Q10 deficiency is a clinically heterogeneous autosomal recessive disorder (23, 24). The phenotypic spectrum associated with mutations in the COQ gene is complex, leading to different phenotypes with mutations in the same gene (12, 25, 26). Gene mutation in neonatal-onset cases have been found to be COQ4 (20/38, 52.6%), COQ7 (2/3, 67%), and COQ9 (6/7, 86%), in addition to COQ2 (11/30, 37%), PDSS1 (1/3, 33%) and PDSS2 (3/7, 43%) (10, 12, 27). COQ4 gene mutation is the most common type in neonatal-onset patients. This study identified a total of 24 cases, with half originating from China. Mullin (17) verified that the missense mutation c.370G>A, p.(Gly124Ser) situated in exon 4 serves as a pathogenic founder mutation in the southern Chinese population. Consequently, it is clear that the prevalence of the mutation within exon 4 is high in the cohort.

The four cases newly reported in this paper all had complex heterozygous or homozygous variants of c.370G>A in COQ4, and they were all located in the Chaoshan region of Guangdong Province. Cases 1 and 2 are compound heterozygote mutations, Mutations in the COQ4[c.300-2A>G(p.?)] locus from the mother were previously unknown. Its impact on protein function is currently unknown, but it could potentially lead to protein truncation or activate the nonsense-mediated mRNA degradation process, which could affect the function of the protein product encoded by the gene. The frequency of this mutation in normal controls is extremely low: G = 0.000008 (2/250938, GnomAD), with pathogenic variants detected at the trans-position (27). Case 3 and 4 are homozygous mutations, Mutations in the COQ4[c.370G>A (p.G124S)], which is included in HGMD, have been observed in multiple patients with primary coenzyme Q10 deficiency and are more prevalent among patients from southern China (16–18). Functional studies revealed that the mutation significantly reduces the expression of the mitochondrial respiratory chain complex II and III (17). Therefore, this locus mutation in southern China is worthy of further study. Xie J et al. (28) discussed the prognosis of COQ4 mutation genotypes from an exon-dependent perspective and believed that different exon regions correspond to different severities. Patients with the East Asian-specific c.370G>A variant show moderate disease severity. In this study, among the 24 patients, 17 were girls, accounting for 70.8% of the total, indicating a higher prevalence rate of girls compared to boys, which highlights an imbalance in the proportion of sexes. Xie J et al. believe that sex is unlikely to be associated with disease severity. Currently, the pathogenesis of COQ4 gene mutation remains clear, and further research is needed to determine if it is related to sex. In comparison to other regions, China, dominated by the c.370G>A mutation, showed no significant difference in mortality rates. Out of the 24 cases, 11 died during the neonatal period, with only 4 surviving. The median survival time was 60 days, suggesting a poor prognosis for neonates with COQ4 gene variants. The depletion of CoQ10 in tissues or cells was first reported in 1989 (15). Typically, when skeletal muscle biopsies indicate a decrease in CoQ10 concentration or reduced constitutive enzyme activity of complexes I + III, and II + III (29), the COQ gene is sequenced. If skin-derived fibroblasts are available, gene rescue experiments or *de novo* CoQ biosynthesis rate measurements can also be conducted using radioactive precursor incorporation (30–32).

The gold standard for determining CoQ10 concentration in biochemical samples is high-performance liquid chromatography (HPLC) (33). CoQ levels in plasma samples obtained by minimally invasive techniques, along with other contents such as leukocytes (lymphoblastoid cell lines or lymphocytes), urine, or skin-derived fibroblasts, have been selected for the measurement (29, 34). The most conventional assay is the measurement of plasma CoQ10 concentration. Its disadvantages include numerous confounding factors and the absence of a normal reference range (35). Interestingly, we observed a significant decrease in plasma coenzyme Q10 concentration(from 4.91 μmol/L to 1.65 μmol/L) in case 4 when the disease worsened due to rotavirus infection. Adjusting the supplemental dose from 20 mg/kg to 50 mg/kg, along with respiratory and circulatory support, led to the successful survival of the child. The potential of using plasma CoQ as an indicator of tissue CoQ deficiency status in patients with mitochondrial disease remains uncertain. Some studies have suggested that monocytes and platelets could serve as alternative indicators. Monocytes are easily isolated from blood, and their CoQ content correlates with skeletal muscle content and is influenced by exogenous supplementation (36, 37). While plasma concentrations of CoQ10 are significantly influenced by dietary uptake (38), the authors (39) focused on blood cells which could be easily isolated from small blood volumes to investigate intracellular CoQ10 concentrations. While excessive environmental supplementation with CoQ10 was without influence on erythrocyte concentrations, a positive correlation exists between plasma content and concentrations in platelets as mitochondria containing cell lines. Compared to plasma, platelet CoQ10 may have important advantages during CoQ10 supplementation (40). The level of CoQ10 concentration in platelets is significantly reduced in mitochondrial disorders such as Parkinson's disease (41). In conclusion, Platelets can be used to assess endogenous CoQ content and to monitor the effects of exogenous CoQ supplementation. Additionally, platelet CoQ content serves as an indicator of mitochondrial electron transport chain function (42). Low-density lipoprotein is the primary carrier of CoQ10 in circulation (43), and plasma CoQ10 status is influenced by circulating lipid concentration. The ratio of CoQ10 to blood cholesterol may help to reflect the true plasma CoQ10 concentration. Measuring CoQ10 levels in cerebrospinal fluid (CSF) is considered one method to evaluate brain CoQ10 status. The preliminary reference range for CSF CoQ10 status is 5.7–9.0 μmol/L (44). Currently, determining blood coenzyme Q10 concentration is the most straighforward method; however, a significant limitation is the absence of a single age-specific reference value or standardized sample preparation and assay method. Moreover, most CoQ10 deficiencies are associated with neurological dysfunction. Therefore, monitoring cerebrospinal fluid and blood concentration during treatment is crucial, and further research is warranted.

Studies (5) have shown that the COQ4 protein palys a key role in CoQ synthesis. COQ4 may serve as a key structural element of the complex, acting as a nucleating factor or scaffolding element during CoQ synthesis. The degree of target organ dysfunction in COQ4 mution cases is different, and we speculate that the mechanism of influence of different exon sites of variation on COQ4 protein is different. On the other hand: it has to do with therapy. Oral CoQ10 supplementation is effective for most children with primary CoQ10 deficiency (13, 45–47). Regarding the treatment of COQ4 deficiency, oral CoQ10 supplements have shown limited benefit both *in vitro* and *in vivo* (26). For responsive patients, the symptoms of multisystem dysfunction, including those affecting the central nervous system (CNS), were improved (23, 33, 48, 49). Administered of CoQ10 at high doses or in specific formulations leads to increased CoQ10 levels in all tissues, such as the heart and brain (50–52). However, some patients with COQ10D7 did not benefit from CoQ10 therapy. Once central nervous system damage is established, recovery is not achievable (13). Apart from variations in formulation and dosage, and the lack of data on *in vivo* CoQ10 levels (28), it remains uncertain from these studies whether the degree of cerebral uptake of CoQ10 would be suffificient to replenish cellular levels of this quinone in a CoQ10 dificiency state (53). A plausible hypothesis suggests that CoQ10 is more effective in preventing further damage rather than reversing existing impairments. The efficacy of CoQ10 therapy appears to be closely linked to the specific COQ4 variants responsible for the condition and the severity of clinical symptoms, as indicated by the limited available data (28).

Factors influencing drug absorption include the formulation, dosage and dosing interval, as well as whether or not a person consumes food during administration. A study conducted by Molyneux (50) provides evidence that a twice-daily dose of 200 mg is more effective in increasing plasma levels compared to a single dose of 400 mg. In terms of absorption, ten 30 mg capsule forms were found to be superior to three 100 mg capsule forms. The dietary intake of CoQ10 was estimated to be very low at approximately 3–5 mg/day (33). Pravst and colleaguest demonstrated that the bioavailability of the water-soluble CoQ10 syrup formulation was 2.4 times higher than that of the ubiquinone capsule formulation (54). CoQ10 is lipophilic, and its absorption is enhanced in the presence of lipids (55, 56). Various strategies have been explored for developing pharmaceutical CoQ10 preparations, including multiple insoluble compounds such as nano-CoQ 10, CoQ10 oil gel, and water-soluble CoQ10 (57). In rodents supplemented with oral CoQ10, high concentrations were observed in several tissues such as liver, ovary, brown adipocytes, and spleen, however, the heart, kidney, muscle, and brain (tissues primarily affected in primary CoQ10 deficiency)did not exhibit significant accumulation (58, 59). Furthermore, a structural analog called β-m-phthalic acid (β-RA), which resembles 4-hydroxybenzoic acid (a precursor of CoQ10 biosynthesis), has shown promise for treating patients with primary CoQ deficiency based on studies conducted using yeast, mammalian cell culture, and mouse models (46). Wang (57) demonstrated that intravenous administration of coenzyme Q10 dissolved in caspofungin microbicide may lead to higher plasma concentrations and thus more effective coenzyme Q10 therapy. Additionally, researchers have proposed modified precursors of the quinone ring structure in CoQ10 as alternative therapeutic options for certain types (58, 60–64). Future therapeutic strategies aimed at augmenting endogenous CoQ10 levels may potentially yield improved clinical outcomes (61, 65, 66).

Wang Y et al. conducted a comprehensive analysis of all studies documenting patients with primary coenzyme Q10 deficiency up until May 2022 (1). No significant adverse effects were been reported. The administered doses varied from as high as 60 mg/day to 2,100 mg/day or as low as 5 mg/kg/day to 100 mg/kg/day, and the duration of treatment ranged from 1 month to 8 years. It has been postulated that CoQ10 treatment may not be able to reverse severe organ tissue damage occurring prior to initiation of therapy (20, 67). However, animal studies utilizing CoQ biosynthetic precursors have demonstrated complete rescue of most phenotypes associated with severe CoQ deficiency through partial CoQ supplementation (58, 61, 62). Partial functional recovery is feasible if the remaining cells and neurons can operate more efficiently by alleviating their deficient state in terms of CoQ10 levels. Furthermore, patients presenting symptoms at a later stage are anticipated to derive greater benefits. Overall, there exists a theoretical advantage even for severely ill patients if substantial amounts of absorbed CoQ10 can reach the affected tissues. In light of the aforementioned speculation, it can be posited that patients exhibiting symptoms suggestive of primary coenzyme Q10 deficiency may benefit from supplementation with coenzyme Q10.

We assume that children with infections, high oxidative stress processes, and hypermetabolic state, such as fever, have an increased demand for CoQ10. Insufficient CoQ10 supply in the body can lead to fatal respiratory and circulatory failure. In Case 4, the administration of a dose of coenzyme Q10 of 50 mg/kg in the acute phase proved to be an effective intervention in reversing the fatal respiratory and circulatory failure. One potential limitation is the relatively low number of confirmed cases. Due to the acute onset and rapid death of neonatal-onset patients and the imperfect detection of mitochondrial function, how gene variants affect the synthesis of CoQ10 still needs further study. Consequently, the statistical significance of the data is constrained by the limited number of cases. Further clinical case studies and data analyses are required. Despite the willingness of families to consent to autopsy, the rate of completion of measurements of mitochondrial complex enzyme activity and tissue coenzyme Q10 concentration remains low. Genetic testing is a crucial tool for diagnosing various conditions, despite the time-consuming nature of the process. A review of population data and case series suggests that COQ4-associated mitochondrial disease may be under-recognized, particularly in the southern Chinese population, where the common pathogenic variant c.370G>A is prevalent. Maria (68) is developing a blood spot test to screen for CoQ10 status during the neonatal period, with the objective of administering CoQ10 treatment at an early stage, before irreversible organ dysfunction occurs (31, 69). Currently, there are a limited number of diagnosed cases globally, and this disease has not yet received sufficient attention. It is recommended that neonatologists, obstetricians, and genetic disease experts in South China enhance their awareness of this disease, which has regional characteristics. It is proposed that the development of a blood spot assay to assay CoQ10 may facilitate the diagnosis of CoQ10 deficiencies during newborn screening and enable treatment in the first few weeks of life. The correlation between blood CoQ10 concentration and the effectiveness of symptom relief remains unknown. Further investigation is required to elucidate the key factors affecting the tissue or cellular uptake of coenzyme Q10. A lack of uniformity was observed with regard to the standardization of CoQ10 dosage, including aspects such as dose, formulation, frequency of administration, timing of initial therapy, duration of therapy, and concomitant medications. It is imperative that more rigorous empirical and clinical documentation be undertaken to fully document the effects of coenzyme Q10 therapy. In conclusion, the pathogenesis of COQ4 gene variation is a challenging area of study.

The majority of previous reports have been individual case studies. This case series report represents an expansion of the number of cases of COQ10D7. A limitation of this study is that many of the cases summarized lack mitochondrial function testing, including the measurement of ETC activities and CoQ10 levels. Despite the initial diagnosis of the COQ4 gene variant in 1989, the challenge of early diagnosis persists. Nevertheless, a synthesis of the cases of COQ4 mutation in the neonatal period revealed striking similarities in the clinical manifestations reported by these patients. It is therefore our contention that this disease is still underrated.

## Conclusion

The primary coenzyme Q10 deficiency is regarded as a clinically heterogeneous disorder and challenging to diagnose condition. It is possible that this disease is underestimated. The prognosis of COQ4 mutation in the neonatal period indicates a low survival rate and an overall poor prognosis. This may be due to the incomplete understanding of the mechanism of how COQ4 gene defects lead to coenzyme Q10 deficiency and why CoQ10 supplementation does not respond well to treatment. The clinical manifestations of neonatal disease are unique, prompt diagnosis and treatment could prolong life expectancy. To improve the diagnostic rate, in addition to genetic testing, mitochondrial functional verification should be prioritized in southern China, where the incidence is relatively high. It will facilitate more in-depth mechanistic studies.

## Data Availability

The original contributions presented in the study are included in the article/Supplementary Material, further inquiries can be directed to the corresponding authors.
