# Supplementary material for: The Spectrum of clinical manifestations in newborns with the COQ4 mutation: case series and literature review
Source: Front Pediatr. 2024 Sep 27;12:1410133. doi: 10.3389/fped.2024.1410133 (PMC11466766; doi:10.3389/fped.2024.1410133)
Supplement: Supplementary file 1 [file Datasheet1.docx]

patient(COQ4 gene c.300-2A>G (p.?) Heterozygous variation, maternal origin)


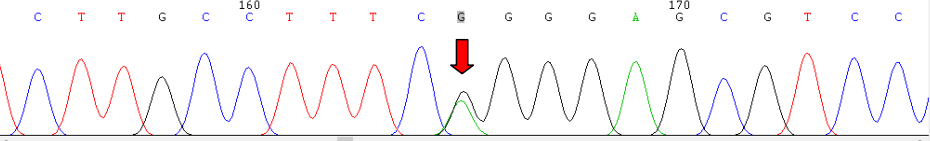


mother


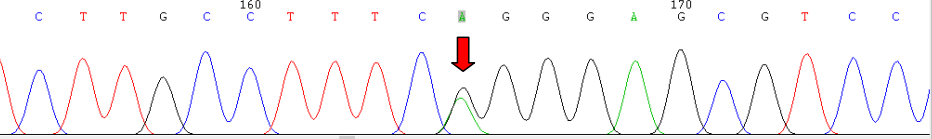


father


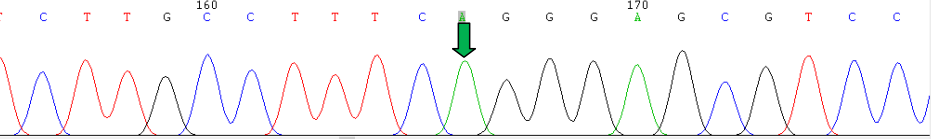


Patient(COQ4 gene c.370G>A (p.G124S) heterozygous variation,paternal origin)


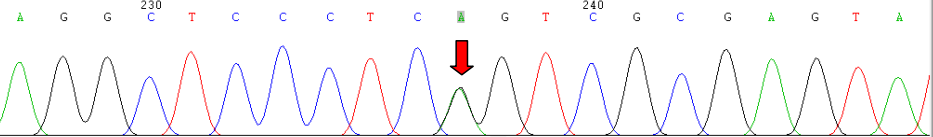


father


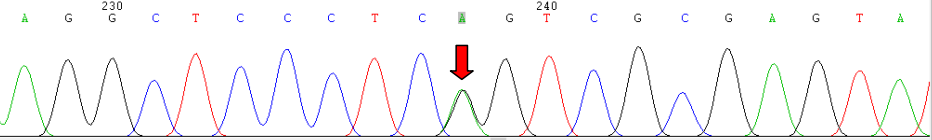


mother


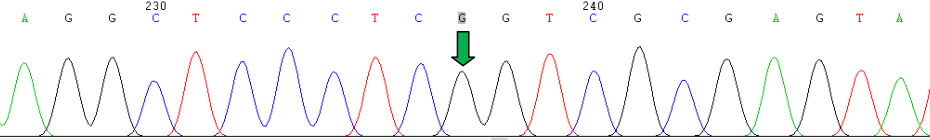


**Fig. 1** Singer sequencing of the COQ4 gene in case 2

Patient(COQ4 gene c.370G>A (p.G124S) homozygous variation)


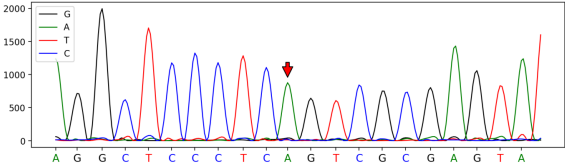


father


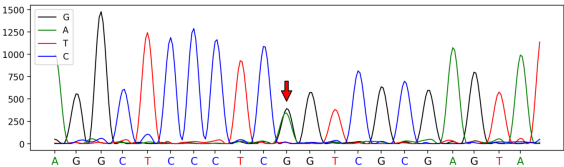


Mother


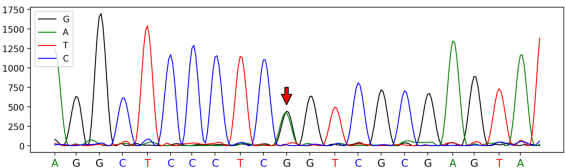


**Fig. 2** Singer sequencing of the COQ4 gene in case 3
